# Droplet Microfluidic-Based *In Situ* Analyzer for Monitoring Free Nitrate in Soil

**DOI:** 10.1021/acs.est.3c08207

**Published:** 2024-01-31

**Authors:** Bingyuan Lu, James Lunn, Ken Yeung, Selva Dhandapani, Liam Carter, Tiina Roose, Liz Shaw, Adrian Nightingale, Xize Niu

**Affiliations:** † Mechanical Engineering, Faculty of Engineering and Physical Sciences, University of Southampton, Southampton SO17 1BJ, United Kingdom; ‡ Department of Geography and Environmental Science, University of Reading, Reading RG6 6AH, United Kingdom

**Keywords:** droplet microfluidics, analyzer, microdialysis, ultrafiltration, *in situ* monitoring, soil nitrate

## Abstract

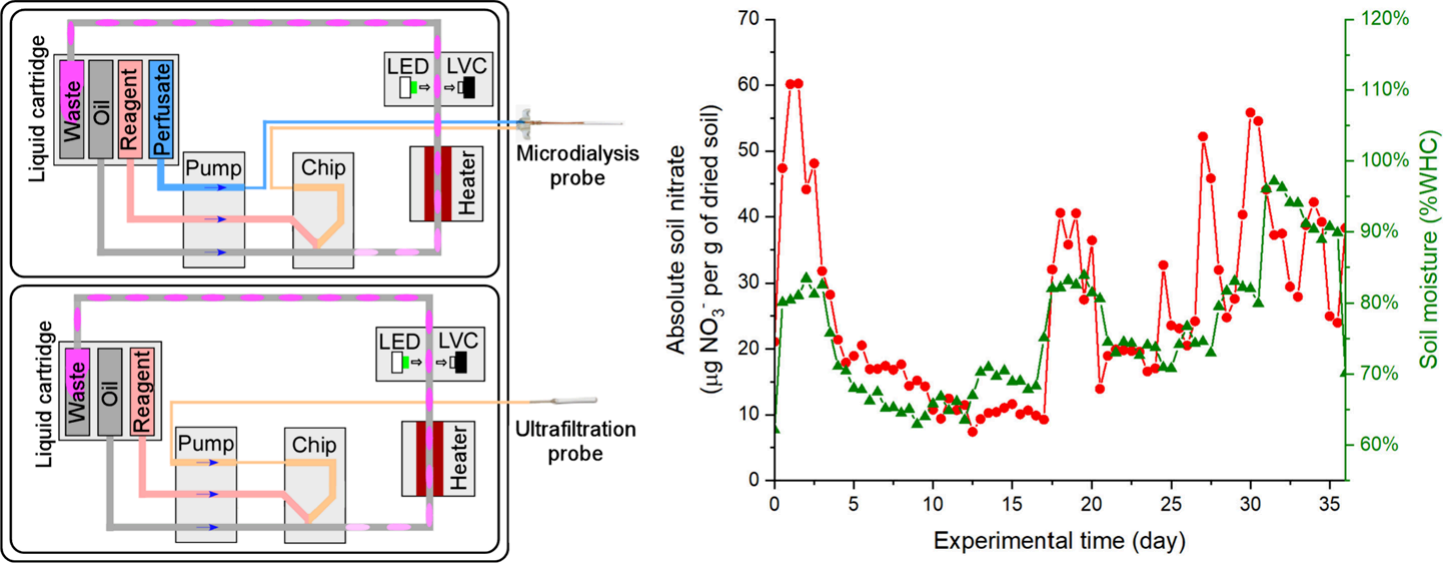

Monitoring nutrients in the soil can provide valuable information for understanding their spatiotemporal variability and informing precise soil management. Here, we describe an autonomous *in situ* analyzer for the real-time monitoring of nitrate in soil. The analyzer can sample soil nitrate using either microdialysis or ultrafiltration probes placed within the soil and quantify soil nitrate using droplet microfluidics and colorimetric measurement. Compared with traditional manual sampling and lab analysis, the analyzer features low reagent consumption (96 μL per measurement), low maintenance requirement (monthly), and high measurement frequency (2 or 4 measurements per day), providing nondrifting lab-quality data with errors of less than 10% using a microdialysis probe and 2–3% for ultrafiltration. The analyzer was deployed at both the campus garden and forest for different periods of time, being able to capture changes in free nitrate levels in response to manual perturbation by the addition of nitrate standard solutions and natural perturbation by rainfall events.

## Abstract 2

A novel in situ analyzer is developed for monitoring free nitrate in soil with potentially wide applications in soil nutrient management and precision agriculture.

## Introduction

Soil is a complex mixture of minerals, organic matter, and organisms, which supports much of life on earth.^1^ In most ecosystems, nitrogen is the soil nutrient limiting plant growth; therefore, the availability of plant-available nitrogen (as dissolved organic nitrogen, nitrate, and ammonium) is key to determining ecosystem productivity.^2^ However, available forms of nitrogen are susceptible to a variety of loss processes (e.g., leaching, ammonia volatilization, and denitrification).^3^ In ecosystems enriched in nitrogen (whether by anthropogenic fertilization or natural atmospheric deposition), these losses can be substantial to cause environmental damages such as pollution of water bodies and climate-impacting emissions of atmospherically active gases. The amount of soil nitrogen in plant-available forms is determined by a complex combination of plant and biogeochemical processes. Monitoring the dynamics of available nitrogen in the soil is needed to provide the insight necessary for managing fertilizer use intelligently in agriculturally managed soils and for understanding the natural ecosystem health consequences of nitrogen enrichment and environmental change.^4^

Soil nitrogen dynamics can be monitored by frequent measurements of soil nitrogen and other soil quality parameters. In response, actions can be taken (e.g., fertilizer addition and irrigation) to optimize plant/crop growth and productivity. Conventional soil analysis involves destructive sampling, sample transfer, nutrient extraction, and lab-based analysis (e.g., using colorimetric assay^5^ and chemiluminescence assays^6^). A standard lab procedure for the analysis of available inorganic nitrogen in the soil consists of extraction of soil with potassium chloride (KCl), removal of the soil by filtration, and analysis of nitrate, nitrite, and ammonium in the filtrate by colorimetry.^7^ Minimal disturbance sampling can also be achieved by ion exchange resins^8,9^ that act like plant roots in absorbing soil nitrogen over time, followed by recovering nitrogen from the resins via the extraction procedure and lab-based analysis. These processes are often time-consuming, laborious, and costly, providing sporadic data hampering decision-making on soil management.

Over the past two decades, many emerging sensor technologies have been developed for continuous monitoring of soil nitrogen *in situ*. The most widely used are electrochemical sensors^10^ composed of an ion-selective membrane and a transducer, which selectively respond to the presence of target molecules. These sensors have been applied to quantification of nitrate,^11^ nitrite,^12^ and ammonium^13^ in soil. Although electrochemical sensors allow rapid analysis without soil pretreatment or use of chemical agents, they suffer from drift over time,^14^ which requires frequent calibration (weekly to monthly) to correct the drifting.^15^ Therefore, they are more suitable for short-term deployment or single measurements than long-term monitoring. An alternative is optical soil sensors based on diffuse reflectance spectroscopy (e.g., near-infrared and middle-infrared spectroscopy),^16^ whereby extensive chemical and physical information (e.g., total nitrogen, total carbon, and moisture content)^17^ can be obtained from spectral analysis of light scattered and diffusely reflected from the soil. Optical soil sensors are nondestructive, environmentally friendly, and can provide fast soil analysis; however, their accuracy is hindered by the variation of sensor-soil distance and the presence of plant residue, stones, and debris.^18^ Hence, there is a need to develop new, quality-assured, and reliable methods that can deliver accurate measurements of critical macronutrients.

Microdialysis, widely used in biomedical research,^19^ has been applied to soil nitrogen analysis in recent years.^20^ Microdialysis probes sample analytes from the soil via passive diffusion through a semipermeable membrane, effectively measuring the diffusive flux of solutes available for plant uptake. Another soil sampling approach is to actively extract pore water from the soil through a filter using a microsuction cup or ultrafiltration probe.^21^ This extracted pore water contains 100% of analytes dissolved, but this method requires soil moisture to be above a set value so that water can be extracted. Both methods can provide high spatial and temporal resolution and cause minimal disruption to the surrounding soil environment. However, currently, these two techniques require long sampling times to collect a sufficiently large sample for the subsequent lab-based analysis,^22^ which is manually intensive and costly.

In this paper, we tackle the challenge of *in situ* and autonomous soil monitoring by designing an integrated analyzer that incorporates droplet microfluidics and microdialysis or ultrafiltration sampling. In our previous work of *in situ* water analysis,^23^ we have demonstrated that droplet microfluidics is an effective tool to miniaturize wet-chemistry-based assays for continuous monitoring of water quality, with advantages of low sample/reagent consumption and non-drifting lab-quality data. Here, we describe how harnessing microdialysis and ultrafiltration sampling techniques can enable nitrate measurement in soil and how measured nitrate can be used to derive the absolute nitrate concentration (μg of NO_3_^–^ per gram of dried weight soil). We show laboratory characterization of the analyzers and their field deployment with an integrated moisture sensor. During deployments, highly dynamic changes in soil nitrate were observed in response to changing conditions, which could not be easily captured by conventional manual and sporadic soil sampling and analysis.

## Materials and Methods

### Materials

Hydrochloric acid (37%), sulfanilamide (≥99.0%), *N*-(1-naphthyl) ethylenediamine dihydrochloride (NEDD, >98%), glucose (≥99.0%), and sodium nitrate (≥99.0%) were purchased from Sigma-Aldrich, UK. Vanadium(III) chloride (≥99.0%) was obtained from Alfa Aesar, UK. Ultrapure water was obtained from a Milli-Q Direct Water Purification System, with a resistance of 18.2 MΩ (Millipore, Merck). Fluorinert FC40 oil was obtained from 3M, UK. The assay used here is a modified Griess reagent method, whereby nitrate is first reduced by vanadium(III) to nitrite, which then reacts with a mixture of sulfanilamide and *N*-naphthyl-ethylenediamine (NEDD) to produce a purple/pink-colored diazonium product,^23^ giving a summation of nitrate and nitrite. For the forest soil sample, the assay mainly quantifies nitrate since nitrite does not usually accumulate in soil.^24^ Preparation of standard solutions and Griess reagent is described in the Supporting Information (Text S1 and S2). All soil used in the lab-based tests was collected from Writtle forest, Chelmsford, UK (51°41′37.9″N, 0°22′20.4″E). The soil detailed information, preparation of nitrate-free soil, standard dried soil, spiked soil columns, and soil characterization (i.e., maximum water holding capacity (WHC%) and conventional analysis) are also stated in Text S3–S5.

### Design of the Droplet Microfluidic System

**Figure 1:**
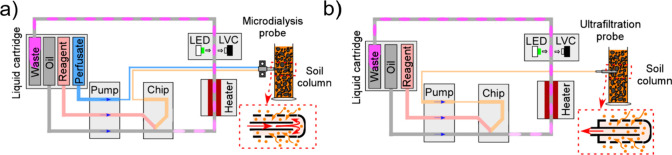
Schematic design of droplet microfluidic units. (a) Sample collection and analysis using a microdialysis probe in soil. (b) Sample collection and analysis using an ultrafiltration probe in soil. The probe sampling diagram was also illustrated for both units, with a red solid arrow showing the direction of flow and orange dots representing free nitrate. The dashed black line indicates the membrane or filter.

Diagrams of the droplet microfluidic system are illustrated in Figure 1, with the system in Figure 1a using a microdialysis probe (CMA8010436, Harvard Bioscience) as the sampling method and Figure 1b using an ultrafiltration probe (no. 19.21.82, Rhizosphere Research Products). Different methods for the installation of probes in soil columns or fields are described in Text S6. Here, a specially designed peristaltic pump, detailed in our previously reported work,^25^ was used to drive the fluidic flows. The roller surface of the peristaltic pump was patterned with grooves to pump the oil and aqueous flows at fixed volumes and sequences but at different phases of the roller rotation. A T-junction droplet microfluidic chip (3D-printed in a Tough PLA material) was connected to the pump outlet. A UT7 PTFE tubing (Adtech Polymer Engineering Ltd., UK) was installed close to the T-junction; therefore, the droplets generated were collected directly into the PTFE tubing to avoid droplet breakup or surface smearing. The reaction temperature (40 °C) in droplets was controlled by an in-house-made heater board. An optical detection flow cell was assembled with an LED light source (535 nm, RS Components Ltd.) and a light-to-voltage converter (TSL257, Farnell) for droplet-based absorption spectroscopy. The working temperature of the flow cell was also controlled at 40 °C by attaching it to the heater to avoid thermal variation.

For sampling using the microdialysis probe, the perfusate (purified water) was pumped through the inlet of the probe (with a flow rate of 3.2 μL/min), and the dialysate with recovered nitrate was introduced to the microfluidic chip (Figure 1a). The sample flow, Griess reagent, and dilution water were mixed into droplets carried by FC-40 oil with one droplet generated every 6 s. The droplets traveled through the heater for 4 min at 40 °C for the reaction to develop, followed by detection within an optical flow cell using absorbance detection.^26^ The absorbance of each droplet was then converted into concentrations (*C*_dialysate_).^20^ Sampling with an ultrafiltration probe, as shown Figure 1b, requires connecting the probe directly to the inlet of the peristaltic pump. The pore water was extracted via the pump into the droplets, yielding a measurement henceforth referred to as *C*_pore_. System calibration was conducted by direct introduction of standard nitrate solutions into the T-junction chip without probes. The sample-to-reagent flow rate ratio was 1:1 for measuring nitrate concentrations below 2 mM. Meanwhile, for the environment with high nitrate concentrations up to 50 mM, an additional pump line was added to dilute the sample with ultrapure water, giving a volumetric flow ratio of 1:2:2 for the sample, reagent, and water, respectively.

The droplet microfluidic system was designed to run in two different modes: continuous running and intermittent running. During continuous running, the peristaltic pump runs continuously at a fixed motor speed and, therefore, at fixed average flow rates. In intermittent mode, the pump was only turned on and run for 30 min at a fixed speed after every 6 or 12 h. Continuous running mode required more reagent and power consumption; therefore, it was only used during system calibration and applications where a rapid change in nutrient levels was anticipated. Meanwhile, intermittent running mode was used for nutrient sampling in all soil-related tests and deployments.

### Integration of the Field Deployable Analyzer

The field-deployable analyzer contained two sets of droplet microfluidic units, each connected to its sampling probe (Figure 5). This allowed the option of either using both sampling methods in a single deployment or using the same sampling method for duplicating measurements. The analyzer was also equipped with a soil moisture sensor (SEN0308, DFROBOT), a timer (DC 12 V-16A, Camway) for scheduled running, a microSD card (Kingston Technology) for data storage, a Teensy 4.1 microcontroller and an interface PCB board, a rechargeable lithium battery (EL12.8-24, Groves Batteries) as power supply, and a 3D-printed liquid cartridge. In the cartridge, aluminum-laminated liquid bags (110 × 180 mm, DaklaPack Europe) were used for storing FC40 oil, purified water, Griess reagent, and waste. The Griess reagent was tested to be stable in the liquid bags for 12 months (stored at 5, 25, and 40 °C respectively) without a discernible change of reactivity. The liquid cartridge was replaced regularly during the monthly maintenance. The used cartridges were brought back to the lab for disposal of chemicals; therefore, the analyzer did not discharge any reagent or waste into the environment. All analyzer components were enclosed in a waterproof box (IP 66, Uriarte Safybox, Spain). The sampling probes and moisture sensor in the soil were connected to the system via a waterproof through hole with a cable gland (RS, UK). The total reagent consumption was about 96 μL per measurement (producing 300 droplets), with an energy consumption at around 16.2 kJ. The liquid cartridge and battery support the analyzer to run for over 30 or 15 days under intermittent running mode with a stop time of 12 or 6 h, respectively. From the current design, the working temperature of the analyzer ranges from 0 to 40 °C. Below 0 °C, the sample inlet could freeze. Above 40 °C, the device will require recalibration as the elevated temperature will increase the reaction speed.

### Derivation of Absolute Soil Nitrate

Here, absolute soil nitrate is defined as the weight of nitrate (μg of NO_3_^–^) per unit of dried weight soil (g). The derivation involves the calculation of external nitrate from measured dialysate (microdialysis) or pore water (ultrafiltration) nitrate via sampling recovery under known soil moisture content and normalization of the unit using the moisture determined at the time of sampling. The correlations between sampling recovery and soil moisture content were determined by quantifying the sampled nitrate (using both microdialysis and ultrafiltration) from a prepared soil column, with a constant spiking concentration (1 mM) and varied moisture content (50–100%WHC). Soil moisture-dependent recovery (*R*_soil_) represents the proportion of measured nitrate concentration from each unit to external nitrate concentration in soil pore water.^27^ This correlation is used to calculate absolute soil nitrate (*C*_soil_, μg NO_3_^–^ per g of dried soil) under known moisture contents via eq 1:
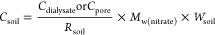
1Here, *M*_w(nitrate)_ is the molecular weight of nitrate (62.0049 g/mol), used for converting the molar concentration (mM) into the weight of nitrate to the volume of water concentration (w/v%). *W*_soil_ is the soil moisture content with units of %WHC, representing the water content (g) per unit of dried soil (g), used for converting w/v% concentration into weight of nitrate to weight of dried soil concentration. The method was validated using the standard dried soil with varied moisture contents, as mentioned in Text S4 and S10.

## Results and Discussion

### Calibration of Droplet Generations and Nitrate Assay in Droplets

As shown in Figure 1, the microdialysis probe collects labile nitrate at the sampling point as soil nitrate diffuses through a semipermeable membrane to the probe as a dialysate (Figure 1a). The ultrafiltration probe acts similarly as conventional suction lysimeters^28^ for extracting pore water through its filter membrane (Figure 1b). For both probes, the collected samples (dialysate and pore water) were encapsulated with reagents into droplets. Before any soil test, the analyzers were calibrated for the continuous generation of droplets by inserting the probes in standard aqueous solutions for 7 days at a frequency of 10 droplets per minute. The average droplet volume was measured to be 0.65 μL on average, with a variation below 3%. We further calibrated the analyzers by the direct introduction of standard nitrate solutions with the sampling probes disconnected. The measured absorbance in droplets is linearly proportional to the nitrate concentration (Text S7 and Figure S1a,b, *R*^2^ at 0.9994) with a LOD of 2 μM, calculated by the 3-sigma method.^29^

### Measuring Nitrate Concentrations from Soil Samples

**Figure 2:**
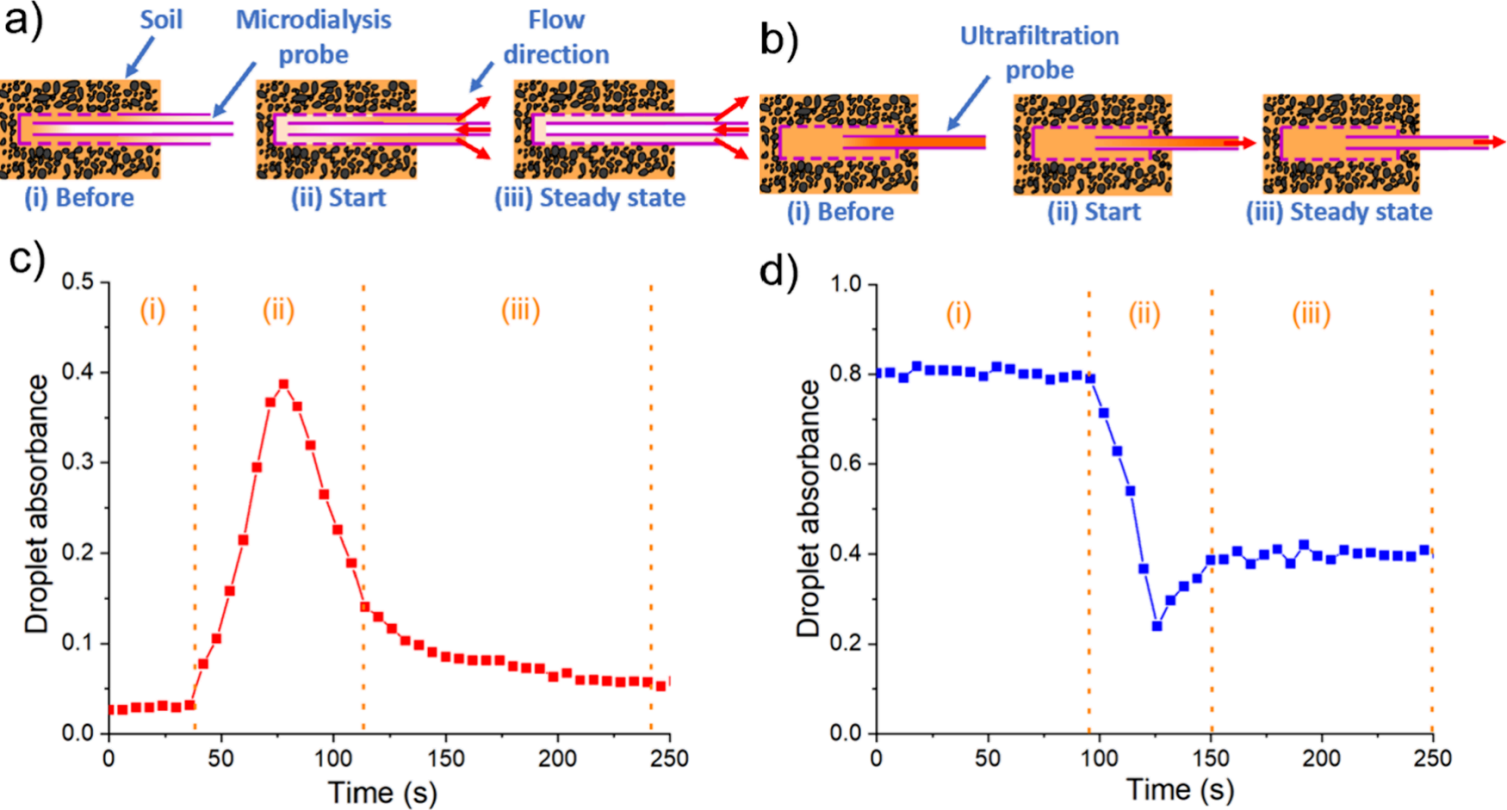
Schematics of sampling mechanisms under the intermittent running mode in (a) microdialysis and (b) ultrafiltration probes. Nitrate in pore water and probes is in orange. Soil is shown as brown particles. The dashed purple line indicates the membrane or filter. The solid purple line represents the probe outline. Flow directions are labeled with a red arrow. Raw absorbance data of each droplet was measured via the detection flow cell (c) from the microdialysis unit and (d) from the ultrafiltration unit.

Early studies using microdialysis as a sampling tool^30^ have demonstrated that continuous sampling from soil can cause the formation of a localized depletion zone around the microdialysis probe, thereby skewing the measurement results. Here, we run the microfluidic systems under intermittent running mode (with 6 or 12 h intervals between running). It is to ensure that at the beginning of each run, the dialysate in the microdialysis probes could reach equilibrium with ambient soil nitrate levels, thereby minimizing the risk of nutrient depletion. Such an intermittent mode of operation brought a unique characteristic transient signal as observed in Figure 2 and was notably different from standard microdialysis method sampling where flow is typically delivered continuously.^30^

Figure 2 shows a typical absorbance response obtained after 12 h stop time when measuring a spiked soil column (1 mM nitrate, 100%WHC). Figure 2a,b illustrates expected molecular movement in the microdialysis and ultrafiltration probes (either by diffusion or bulk fluid movement), and Figure 2c,d shows the corresponding measured absorbance. A full set of data from Text S8 and Figure S2 shows the sequence of droplets generated from previous measurements, residual liquid samples, and fresh samples in the fluidic conduit.

When sampling with a microdialysis probe after a stop time, there are three expected phases of analyte concentration change (Figure 2a). Before the pump restart, the tubing downstream of the probe contains residual dialysate (Text S8 and Figure S2a,b) from previous measurements (Figure 2a-i,c-i). As the fluid has been stationary for 12 h, there has been sufficient time (Text S9 and Figure S3) for liquid on either side of the membrane at the probe tip to reach equilibrium; hence, the fluid within the microdialysis tip has a comparable nitrate concentration to the fluid immediately outside. When the pump restarts, the fluid within the probe moves out of the probe, causing an increase in measured nitrate (Figure 2a-ii,c-ii). The measured absorbance sees a high peak as the initial dialysate comes through. After the steady state has been established, the dialysate will contain a much lower nitrate concentration as the residence time at the membrane is much shorter (around 6 s), which is insufficient for the fluid across the membrane to reach equilibrium, leading to lower measured nitrate (Figure 2a-iii,c-iii). Note that the observed peak is a smooth rather than a square wave (Figure 2c-ii) due to Taylor dispersion in the continuous flow section before the droplets are generated.^31^ As the absorbance peak closely resembles the actual concentration outside of the tip, and it provides a much stronger signal relative to the steady state (and, hence, higher sensitivity), we use this peak value as the characteristic point for nitrate quantification.

Figure 2a,c shows that during steady-state operation, the absorbance (nitrate concentration) decreases over time, which is consistent with previous reports of soil measurement, where the presence of solid matter and the connectivity of moisture inhibit the transport of nitrate to the probe. When this transport is lower than the removal by the probe, the nitrate concentration around the probe is gradually reduced, creating a so-called “depletion zone”.^30^

The representative measurement from an ultrafiltration probe is shown in Figure 2b,d. Before the pump starts, all channels downstream of the probe contain a residual sample (Figure S2c,d) from the previous measurement (Figure 2b-i,d-i). When the pump is activated, pore water is drawn into the probe after a short transition (Figure 2b-ii,d-ii) to a steady state where the fresh sample (with lower nitrate concentration) is continuously drawn in and measured (Figure 2b-iii,d-iii). In practice, we took the average absorbance of the first 12 droplets in the steady state for the ultrafiltration measurement. The pattern of measurement from the ultrafiltration probe is much simpler than the equivalent microdialysis measurement due to the differences in how samples are obtained: forced liquid extraction versus diffusive extraction.

With the absorbance measurement from each sample obtained, the nitrate concentrations in the droplet can be calculated via the pre-calibration with known nitrate solutions (Figure S1). However, this concentration is not equivalent to the absolute nitrate concentration in the soil, as the recovery rate to the sampling probes from the soil is affected by different soil moisture contents, which will be further discussed in the following section.

### Nitrate Recovery Study under Different Soil Moisture Contents

In principle, both sampling methods can be affected by the moisture content of the soil: In ultrafiltration, pore water samples are pulled into the probe by negative pressure; therefore, air can be pulled into the probe in unsaturated soil. Microdialysis, meanwhile, is dependent on diffusion pathways. At lower moisture content, the soil will possess reduced diffusion pathways, thereby restricting the recovery rate of the microdialysis.

To study the relationship between soil moisture and nutrient recovery relative to the absolute soil nitrate (in μg of NO_3_^–^ per g of dried soil, as normally measured by KCl extraction), an experiment was carried out by measuring recovered nitrate from soil columns containing nitrate-free soil plus 1 mM standard nitrate and varied moisture from 50 to 100%WHC.

**Figure 3:**
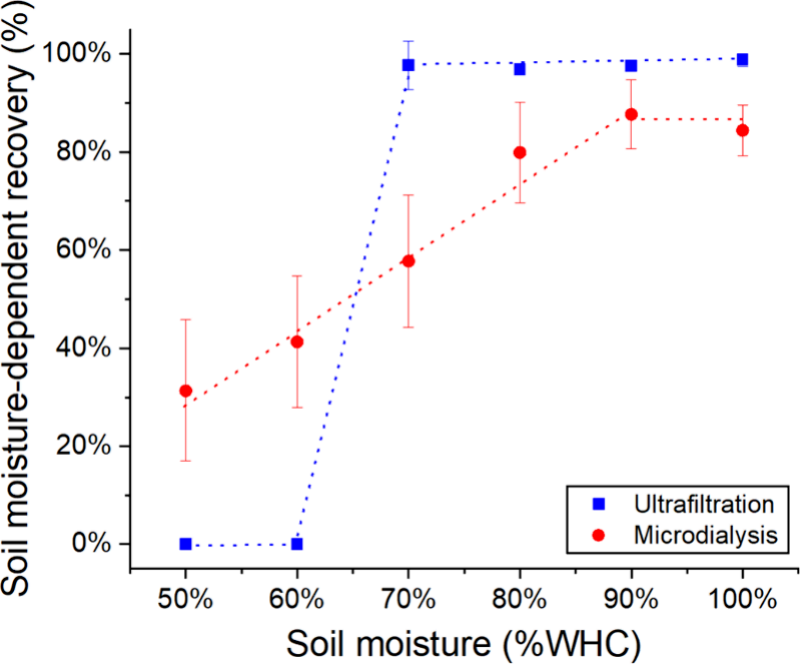
Nitrate recovery under different soil moisture contents from 50 to 100%WHC, measured from either the microdialysis (red circle) or ultrafiltration (blue square) unit under 12 h stop time. The error bars correspond to the standard deviation of calculated recovery from three replicates measured from three soil columns.

Figure 3 plots the soil moisture-dependent recovery against soil moisture content under 12 h stop time from each sampling method. The recovery is defined as the measured concentration relative to the concentration of the added standard solution. For microdialysis, the recovery reaches its maximum at 83–87% for WHC above 90% but does not reach full recovery. This is due to Taylor dispersion from the continuous flow section of fluidics before droplet generation, as discussed earlier. When the soil moisture is lowered to 50%WHC, the recovery is reduced linearly to 31%.

The recovery from the ultrafiltration probe remained consistently above 95% when soil moisture content was 70%WHC or above. It is also noticeable that the ultrafiltration measurements showed much less error than microdialysis (as shown by the error bars), as ultrafiltration no longer relies on the diffusion of nutrient molecules across the membrane and surrounding soils. At moisture levels below 70%WHC, however, the measured nitrate via the ultrafiltration probe immediately dropped to zero, and air bubbles were observed in the tubing. This was due to air being pulled in preferentially to water, consistent with a previous report where a similar suction-based sampling method was performed.^32^

The soil moisture-dependent recovery in Figure 3 shows that ultrafiltration features constant and full recovery as it directly measures pore water, but it can only operate at high moisture levels. On the contrary, microdialysis sampling can work under a much wider soil moisture range but usually cannot provide full recovery. Here, it is interesting to note that Brackin et al. suggested that microdialysis measurement represents more closely the amount of nitrate available to plant roots,^33^ taking into account nutrient transports.

Using eq 1, we further calculated the LODs of absolute soil nitrate for each method at the maximum recovery. Since the LOD of the flow cell when standard solutions were fed directly into each analyzer was 0.002 mM (i.e., obtained without a sampling probe), the LOD of *C*_soil_ from the microdialysis unit was around 0.07 μg NO_3_^–^ per g of dried soil (calculated from 83% recovery at 100%WHC), and that of the ultrafiltration unit was 0.06 μg NO_3_^–^ per g of dried soil (calculated from 99% recovery at 100%WHC).

The recovery rates in Figure 3 can be used to estimate the absolute soil nitrate (*C*_soil_) from measured *C*_dialysate_ or *C*_pore_, which was validated using standard dried soil spiked under various moisture contents with high accuracy, as described in Text S10 and Figure S4. It should be noted that these calibrations are valid only for the type of soil used for calibration. Different soils have different texture, porosity, composition, and other geophysical properties. Variations in all of these properties will influence the performance of sampling probes, especially the recovery of the microdialysis probe. Therefore, to apply the analyzer to different types of soil, recalibrations are required to follow the procedures discussed earlier.

### Monitoring Nitrate in the Laboratory Soil Column

**Figure 4:**
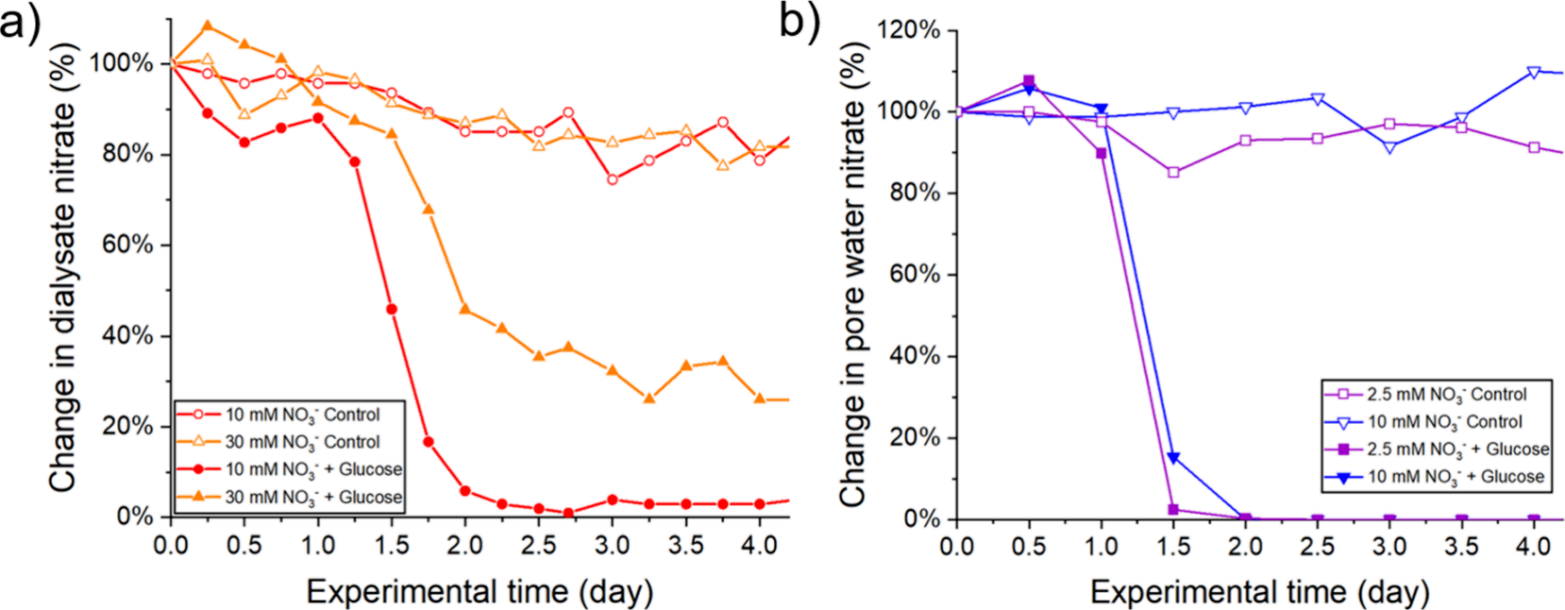
(a) Change of dialysate nitrate in the soil column measured from a microdialysis unit. (b) Change of pore water nitrate in the soil column measured from ultrafiltration units. Soil columns were spiked with standard nitrate solutions (2.5, 10, or 30 mM) with 100 mM glucose (solid symbol) or without glucose (as control, hollow symbol). Each point is a single measurement obtained from a microdialysis or ultrafiltration unit.

We applied the droplet microfluidic unit in a controlled laboratory experiment to determine whether it could capture dynamic nitrate concentration changes. Since the addition of a labile carbon-rich substrate (e.g., glucose) to the soil will likely serve as a carbon and energy source to accelerate microbial nitrate immobilization^34^ or pathways for dissimilatory reduction of nitrate,^35^ we monitored nitrate concentrations from soil columns with various amounts of nitrate added. These soil columns were prepared with the same moisture content (at 100%WHC) and initial nitrate levels (by using nitrate-free soil and adding standard solutions (2.5, 10, or 30 mM). Figure 4 shows the change of dialysate and pore water nitrate observed over 4 days.

Both sampling methods captured similar trends: For soil spiked with glucose, a sharp drop in soil nitrate was monitored between day 1 and day 2, consistent with expectations.^36^ The magnitude of the drop varied depending on the starting nitrate concentration, with nitrate dropping to almost zero for th10 mM added glucose and ∼70% drop for a higher starting nitrate concentration (30 mM) (Figure 4a). At high concentrations, nitrate likely exceeded the capacity of microbial consumption within this short period, which was consistent with those in similar previous experiments.^37^ The ultrafiltration data (which used starting nitrates of 2.5 and 10 mM) showed very similar behavior to the 10 mM microdialysis data, with nitrate dropping sharply on day 2 to below the LOD (Figure 4b). For the controls where no glucose was added, nitrate only reduced slightly over time, indicating limited consumption of nitrate without glucose supply. Overall, these results showed that both sampling methods were consistent with each other and could be used to monitor and characterize dynamic changes of nitrate in soil.

### Field Deployment 1 (Campus Garden)

**Figure 5:**
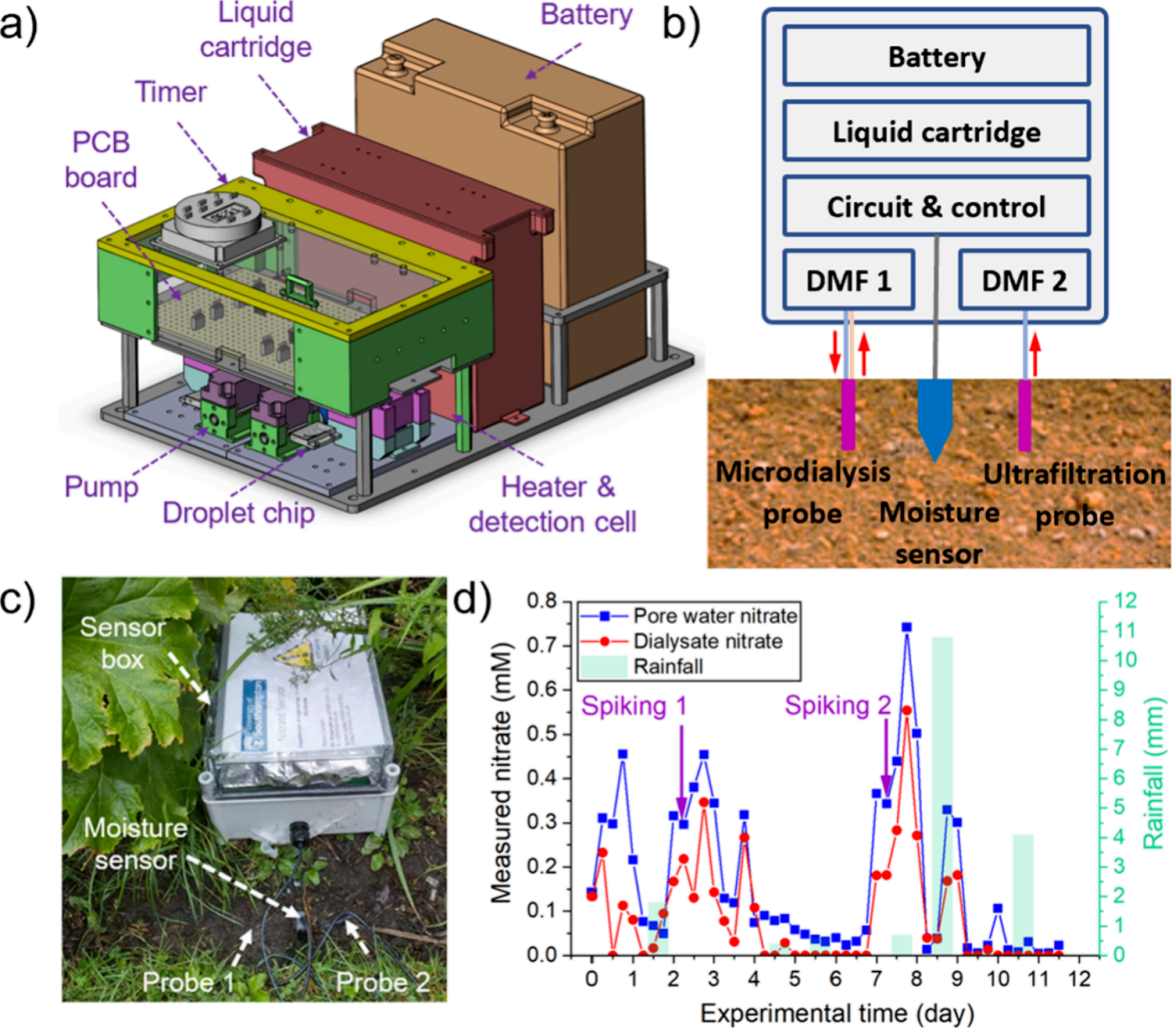
Analyzer field deployment in the campus garden of the University of Southampton, (a) 3D schematics of the field-deployable analyzer, including components such as control board, timer, liquid cartridge, battery, and fluidic system. (b) 2D schematics for the analyzer integrated with sampling probes and moisture for *in situ* soil monitoring. (c) Photo of the deployment location and targeted campus soil, located close to a local stream. (d) Change in dialysate nitrate (microdialysis, red cycle) and pore water nitrate (ultrafiltration, blue square) over the 12 day running in the field. Rainfall was recorded as light green bars in the graph. Experimental time: day 0 is 21st September 2022 and day 11 is 3rd October 2022. Each point is a single measurement obtained from the microdialysis or ultrafiltration unit.

Having shown that the analyzer could monitor dynamic changes in soil nitrate, we then moved to *in situ* deployments. Initially, we targeted a local deployment on the university campus with manual perturbations to the nitrate concentrations by spiking with standard nitrate solutions to ensure that there were observable and predictable changes. The analyzer design with key components (i.e., fluidic, electron, and supply systems) is illustrated in 5a,b. The analyzer comprised two droplet microfluidic units with one connected to a microdialysis probe and the other to an ultrafiltration probe. These two sampling probes were inserted 10 mm deep into the soil (Figure 5c) along with a moisture sensor. Together with the analyzer, the moisture sensor was also calibrated in a lab-scaled soil column (50–100%WHC), as explained in Text S11. The linear relationship was used to determine the soil moisture at the sampling time (Figure S5). However, the reading of the moisture sensor plateaued at its maximum analogue to digital reading and did not change during the 12 day test, indicating that the testing soil had high moisture content. The analyzer ran autonomously at 8 am/pm and 2 am/pm to give four nitrate measurements per day from 21st September 2022 to 3rd October 2022. The nitrate concentrations in dialysate (microdialysis) and pore water (ultrafiltration) are plotted in Figure 5d, together with spiking and rainfall events.

Overall, changes in pore water and dialysate nitrate showed similar trends, with nitrate levels responding to rainfall and the manual addition of nitrate. A very low nitrate level (0.15 mM pore water nitrate and 0.13 mM dialysate nitrate) was measured on day 0 (Figure 5d). Nitrate standard solutions (1 mM on day 3 and 2 mM on day 8) were added to mimic fertilization of fast-release mineral nitrate into the soil. Both dialysate and pore water nitrate immediately increased after spiking. The magnitudes of the peaks on day 3 (0.45 mM pore water nitrate and 0.35 mM dialysate nitrate) and day 8 (0.74 mM pore water nitrate and 0.55 mM dialysate nitrate) were commensurate with the concentration of the nitrate spiking solutions administered beforehand.

Following these spiking-induced peaks, the nitrate levels dropped sharply, indicating immediate nitrate leaching through drainage water under saturated moisture content or potential rapid denitrification and uptake by the plant nearby. Rain events on days 1 and 8 might also have contributed to the increase of available nitrate in soil, as rainfall could enhance nitrate transport.

Over the 12 day autonomous running, 47 measurements were carried out without any maintenance to the analyzer. Due to the constant high moisture content in the soil, both ultrafiltration and microdialysis units worked consistently without any observed depletion or air bubble formation in the microfluidic system.

### Field Deployment 2 (Writtle Forest)

**Figure 6:**
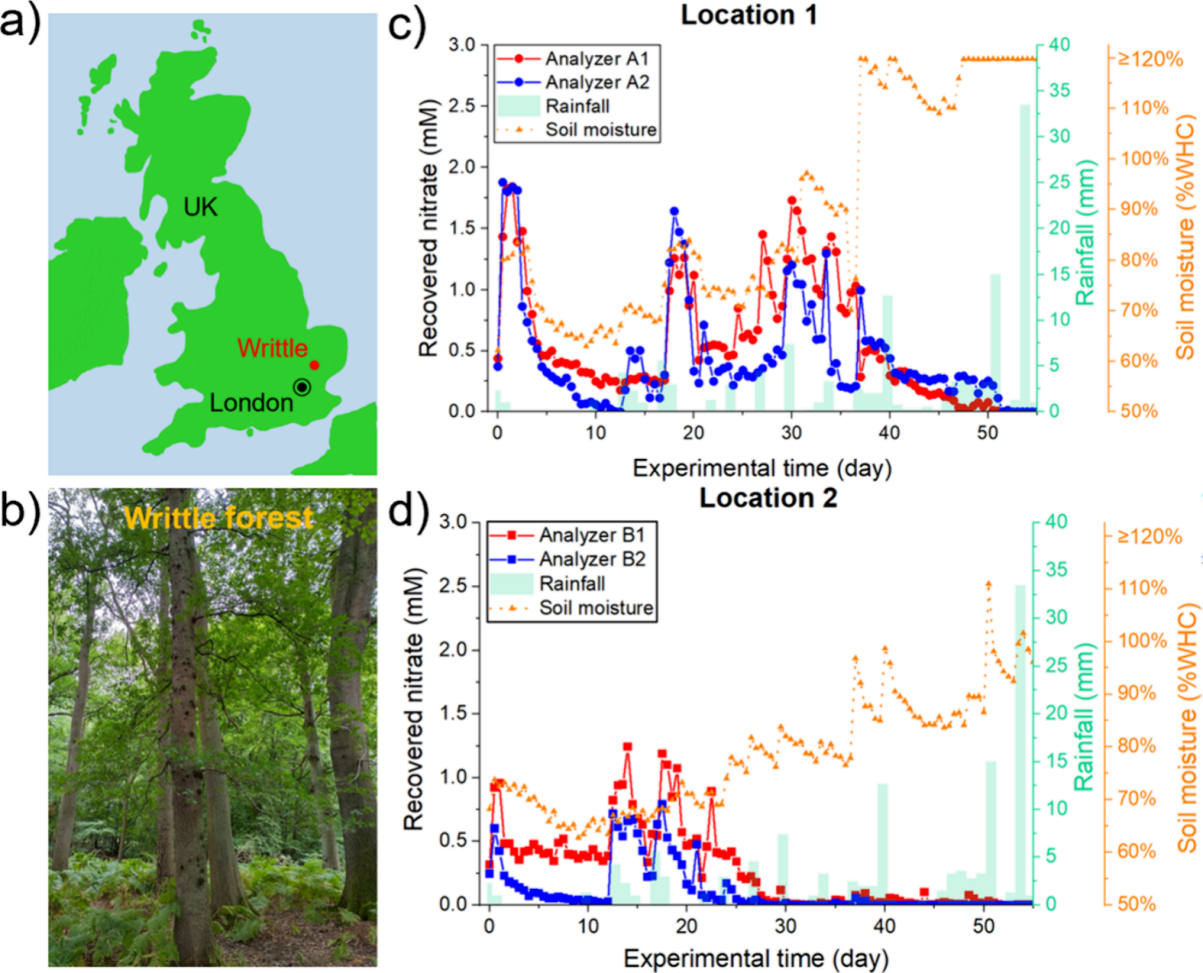
Field deployment in Writtle Forest. (a) UK map showing the location of Writtle forest, at north London, near Chelmsford. (b) Photo of Writtle woodland forest with oak trees. (c, d) Recorded change in dialysate nitrate over a month of deployment at locations 1 and 2, respectively. Soil moisture measured from the moisture sensor was plotted as an orange triangle in a dashed line. Rainfall was recorded as light green bars in the graph. Experimental time: day 0 is 14th September 2022 and day 62 is 15th November 2022. Each point is a single measurement obtained from the microdialysis unit.

Following the initial deployment at the campus garden, two field analyzers (Analyzer A and Analyzer B) were deployed in Writtle forest (map shown in Figure 6a), an ancient semi-natural woodland (Figure 6b) comprised of mature oak trees (mainly *Quercus robur*) in mixture with other tree species such as Hornbeam (*Carpinus betulus*). As the deployment (14th September to 15th November 2022) started after a protracted dry summer period in July and August 2022, the soil was judged to be too dry for the ultrafiltration probe. Each field analyzer was equipped with two microdialysis units (A1, A2 and B1, B2), along with a moisture sensor (calibrated in a lab-scaled soil column, Figure S5). The probes of A1 and B1 were embedded in the soil, while A2 and B2 were using a needle-based method for comparison, as described in Text S6. The analyzers were set under two different oak trees, each approximately 1.5 m away from the tree stem, and ran 30 min from 8 am and 8 pm, giving one measurement of dialysate nitrate every 12 h. Maintenance of the analyzers was performed once every month, consisting of battery and reagent replacement. The analyzers were also calibrated on-site using standard nitrate solutions both before and after deployment. The calibrations showed only less than 4.7% variation during the whole deployment period, indicating the high robustness of the analyzer for long-term deployment.

Over 62 continuous days, 124 nitrate measurements were obtained from each unit. Figure 6c shows the measured nitrate concentrations and soil moisture from Analyzer A in addition to local rainfall (temperature data shown in Text S12 and Figure S6). In the first 40 days, we observed dynamic changes in nitrate concentrations, with a regular pattern of rainfall producing rises in measured nitrate, which subsequently dropped after a day or so. The rainfall also resulted in an accumulative increase in soil moisture levels. From days 40 to 50, the measured nitrate concentration reduced gradually to lower than 0.2 mM, coinciding with a sharp rise in moisture levels after day 35. The nitrate further decreased to an undetectable level after two heavy rain events on days 50 (15 mm) and 54 (33 mm).

The nitrate data from the two units within the analyzer were both qualitatively and quantitatively consistent, with similar rainfall-related peaks on days 1, 18, 30, 34, and 37, similar short-term drops during dry periods, and a longer-term drop as the soil became saturated. Small variations between the two units are likely due to the slight difference in location and different probe–soil interactions resulting from the two probe installation methods.

Location 2 (Analyzer B) showed similar behavior (Figure 6d): short-lived boosts in nitrate levels from rainfall (days 1, 14, 18, and 22) paired with short-lived drops during dry periods at the start of the deployment and then a decrease to near zero after day 30. Once again, the two units within the analyzer gave very consistent data. Compared with location 1, the long-term drop in nitrate levels and high moisture occurred earlier at location 2. This is likely due to the lower initial nitrate levels in the soil at location 2, which were more easily diluted or leached by intermittent heavy rains. An additional notable difference was that the soil moisture was more dynamic in location 1 than in location 2. The soil moisture sensor plateaued after heavy rain on days 37, 40, and 48 in location 1 (Figure 6c); however, the moisture sensor in location 2 could still capture changes after day 37 (Figure 6d). These differences could result from differences in canopy cover or in drainage at each location. While the change of recorded temperature (Figure S6) from the weather station fluctuated during the deployment, no obvious correlation was observed between temperature nitrate levels.

**Figure 7:**
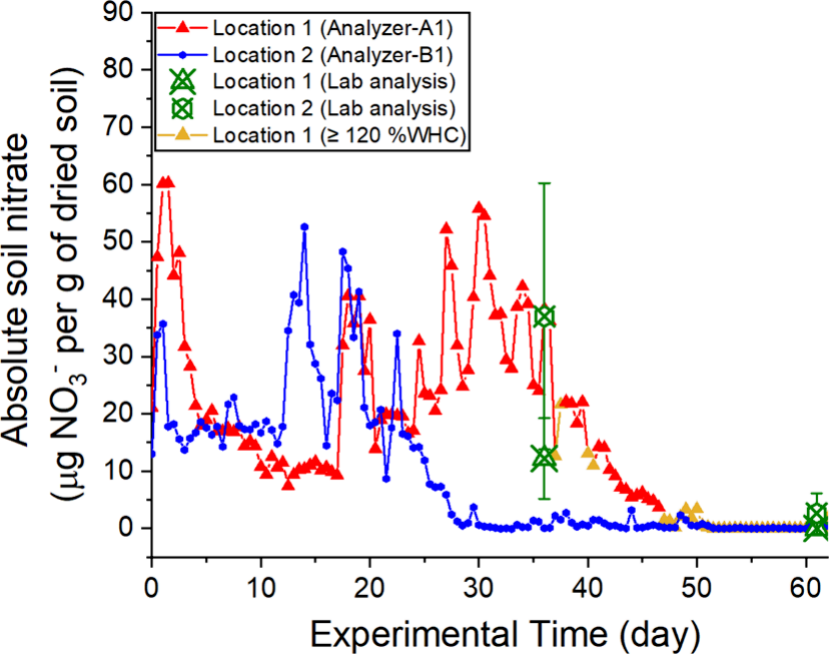
Absolute soil nitrate (filled markers and solid lines) calculated from the corresponding soil moisture-dependent recovery and dialysate nitrate from Analyzer A1 and B1. Triangle markers for location 1 and blue circle markers for location 2. Where soil moisture exceeded the moisture sensor detection limit (≥120%WHC) in location 1, the triangle markers are colored yellow rather than red. Experimental time: day 0 is 14th September 2022 and day 62 is 15th November 2022. Each point is calculated from a single measurement obtained from the microdialysis unit. Manual samples at similar locations, later analyzed in the laboratory, are shown by the hollow green markers: the hollow crossed triangle and circle markers correspond to soil nitrate measured from samples collected at locations 1 and 2 around the analyzers. Error bars represent the standard deviation from the three replicates.

Using the correlation between recovery and moisture content (Figure 3), the absolute soil nitrate was calculated using eq 1, and it is shown in Figure 7. As the recovery plateaued above 100%WHC (Figure 3), in cases where the soil moisture levels were above 100%WHC, plateau recovery (average recovery of 85% at 90–100%WHC) was used. Where soil moisture exceeded the upper detection limit of the moisture sensor (≥120%WHC on days 37 and 38 and after day 47, shown by yellow triangle markers in Figure 7), the absolute soil nitrate was calculated assuming a soil moisture content, *W*_soil_, of 120%WHC.

The nitrate level at location 1 varied between 1.8 and 60.2 μg of NO_3_^–^ per gram of dried soil before it dropped down after heavy rain on day 50. Location 2 had a lower nitrate level from 0.1 to 52.7 μg of NO_3_^–^ per gram of dried soil, which decreased close to 0 after day 30. Soil samples were collected on days 36 and 61 around each deployment location, and conventional lab-based wet-chemistry analysis (described in the Supporting Information) was carried out with data shown in Figure 7 (lab analysis). The data from manually collected samples are generally consistent with that of *in situ* monitoring showing nitrate levels initially being in the range of tens of μg of NO_3_^–^ per gram of dried soil (day 36) before dropping to zero or near-zero levels later in the deployment (day 62). Overall, Figure 6 shows a highly dynamic change in soil nitrate for both locations, which could be significantly influenced by rainfall and other environmental changes. Note that a similar reduction of soil nitrate from September to November was also reported in early studies.^38,39^ It should be noted that soil is notably heterogeneous^40^ and the manual samples show large intersample variability, as shown in the large error bars in Figure 7.

Similar to dialysate nitrate, absolute soil nitrate in location 1 is much higher than that in location 2. This difference could be due to the non-uniform distribution of nitrate in the soil, different levels of uptake from oak trees’ roots, or varied consumption of nutrients through microbial activity.

These field deployments have demonstrated that droplet microfluidic analyzers were capable of *in situ* and real-time monitoring of soil nitrate dynamics. For microdialysis sampling, we have developed a method of deriving absolute soil nitrate, which allowed a direct comparison of the data from the analyzer with conventional lab analysis. While ultrafiltration sampling is more suitable for fields with higher soil moisture contents (≥70%WHC), the microdialysis sampling method was shown to be more robust for a wider range of soil moisture contents (≥50%WHC). Noted monitoring with a single probe can only give information for a very small and specific location where the probe is located. Comprehensive information for a large area (e.g., an agricultural field) may require measurements from different locations, which can be achieved by using multiple probes/analyzers or a combination of the analyzer and other sensors or conventional pooled soil sampling methods.

## Supporting Information Available

Standard solution preparation, Griess reagent preparation, soil information and preparation of nitrate-free and standard dried soil, preparation of lab-scaled soil columns, soil characterization, installation of sampling probes, pre-test sensor calibration without probes, full droplet absorbance response over time, soil moisture-dependent recovery under varied stop time, method validation on standard soil, moisture sensor calibration, and forecast temperature during Writtle forest deployment (PDF)
