# Supplementary material for: Droplet Microfluidic-Based In Situ Analyzer for Monitoring Free Nitrate in Soil
Source: Environ Sci Technol. 2024 Jan 31;58(6):2956–65. doi: 10.1021/acs.est.3c08207 (PMC10867830; doi:10.1021/acs.est.3c08207)
Supplement: Supplementary file 1 — es3c08207_si_001.pdf [file es3c08207_si_001.pdf]

Supporting information

Droplet microfluidic-based *in situ* analyzer for monitoring free nitrate in soil

Bingyuan Lu,[1] James Lunn,[1] Ken Yeung,[1] Selva Dhandapani,[2] Liam Carter,[1] Tiina Roose,[1] Liz Shaw,[2] Adrian Nightingale,[1] and Xize Niu[1]*

1. Mechanical Engineering, Faculty of Engineering and Physical Sciences, University of Southampton, Southampton, SO17 1BJ, United Kingdom

2. Department of Geography and Environmental Science, University of Reading, RG6 6AH, United Kingdom

Corresponding author: Prof Xize Niu, x.niu@soton.ac.uk

Number of pages: 7

Number of texts: 12

Number of figures: 6

Text S1：Standard solution preparation

To prepare a 50 mM stock solution of sodium nitrate, 0.425 g of sodium nitrate was dissolved in water in a 100 ml volumetric flask. Serial dilution of the stock solution was performed to generate standard nitrate solutions ranging from 0.1 to 50 mM. Furthermore, a 100 mM glucose solution was prepared by dissolving 9 g of glucose in water in a 500 ml volumetric flask.

Text S2：Griess reagent preparation

Griess reagent was made by first adding vanadium (III) chloride (1.25 g) to 50 mL ultrapure water. Hydrochloric acid (15 mL, 37%) was then added, followed by sulfanilamide (1.25 g) and N-1-naphthyl ethylenediamine dihydrochloride (NEDD, 0.125 g), before being shaken until all solids were dissolved. The solution was then made up to 250 mL with water, forming a turquoise color, and was then stored in capped aluminum-lined bags to prevent oxidation and stored at 4°C before use.

Text S3：Soil information and preparation of nitrate-free and standard dried soil

The Writtle soil was with 13.54 % organic matter (determined by ignition at 500 °C) after sieving to particle size below 1.4 mm and pH around 4 (measured with 5 g soil in 12.5 ml ultrapure water). It has a maximum water holding capacity (%WHC, $w_{(water)}/w_{(dry\ soil)}$%) of 0.5 g of water per g of dried soil (on the basis of oven dry 105 °C), determined by the method stated in the following section. Nitrate-free soil was prepared by first mixing Writtle soil (150 g) with purified water (1.5 L). After 15 min, the mixture was centrifugated to remove the aqueous supernatant from the soil suspension. The process was repeated for six times to remove detectable nitrate in the soil. The rinsed soil was then dried in an oven at 60°C for 72 hours. Standard dried soil (around 15 μg NO_3^- per g of soil) was also made by mixing 200 g of nitrate-free Writtle soil with 100 ml of 0.5 mM standard nitrate (as $NaNO_3$) solution, and then dried and sieved via the same procedures.

Text S4: Preparation of lab-scaled soil columns

Lab-scaled soil columns were made up in 30 ml sample vials (2 cm in diameter), with holes (2 mm in diameter) drilled on the side for insertion of sampling probes. Vials were first filled with dried soil (6.67 g in total), followed by the addition of aqueous solutions with variable volume (1.67 to 3.35 ml) in order to control the soil moisture content from 50 to 100 %WHC, respectively. Four different combinations of soil columns were prepared: 1) nitrate-free soil + 1 mM standard nitrate solution, 50 to 100 %WHC; 2) standard dried soil + purified water, 50 to 100 %WHC; 3) nitrate-free soil + standard nitrate solution (2.5, 10 or 30 mM), 100 %WHC; 4) nitrate-free soil + standard nitrate solution (2.5, 10 or 30 mM) + 100 mM standard glucose solution, 100 %WHC. Soil columns 1 and 2 were used for initial analyzer calibration and method validation respectively. Soil columns 3 and 4 were tested for short-term nitrate monitoring in lab.

Text S5: Soil characterization (SI)

The soil used in all lab-based experiments was collected from Writtle forest, England. To determine its maximum water holding capacity, fully saturated soil was prepared by inserting soil (approx. 5g) into a container with a meshed bottom and placing it into a dish of water. After 6 hours, this container was then suspended above the dish to allow excess water to drain. The drained soil was transferred to a crucible and weighed, then the filled crucible was placed in the oven at 105°C for at least 24 hours or until no further water loss occurs. The maximum water holding capacity was then calculated by:

$$\% \, WHC = \frac{mass\ of\ drained\ soil - mass\ of\ oven\ dried\ soil}{mass\ of\ oven\ dried\ soil} \quad \text{x } 100\%$$

For conventional lab-based soil analysis, 40 g of soil was mixed with 200 ml of 1 mM KCl solution. The mixture was shaken for 1 hr and then filtered through a GF/A filter paper. The Skalar wet chemistry analyzer (Skalar Analytical B.V., Netherlands, with limit of detection (LOD) at 2.24 μg N per g of dried soil) was used to determine the nitrate in the filtrate *via* the hydrazine reduction method. Nitrate is reduced to nitrite by hydrazinium sulfate and the nitrite (originally present plus reduced nitrate) is determined by diazotizing with sulfanilamide and coupling with N-(1-naphthyl) ethylenediamine dihydrochloride to form a highly colored azo dye which is measured at 540 nm.

Text S6: Installation of sampling probes

Three methods were used for installation of sampling probes into soil. Method 1: For lab-scaled soil column, a needle was used to punch a hole through the 2 mm side hole on the sample vial. Probes were then inserted. Method 2: For field deployment, surface soil (10 cm) was partially removed and then a rectangular groove (around 10 x 3 x 3 mm) was made on the soil surface using a blade. The probe was embedded in the groove then covered with removed surface soil. The probes were placed 10 cm away from each other to avoid potential probe to probe interaction. Method 3: For field deployment, after removal of surface soil (10 cm), a needle was used to punch through the soil surface to form a hole and then sampling probe were gently inserted into the hole, followed by covering back the surface soil.

Text S7: Pre-test sensor calibration without probes

Prior to conducting soil experimentation, a preliminary test of the droplet microfluidic unit was performed to establish the relationship between nitrate concentration and measured absorbance without the use of a microdialysis or ultrafiltration probe. This was achieved by directly introducing standard nitrate solutions ranging from 0.1 to 2 mM into each system and establishing a linear correlation, as shown in Figure 1. The error bars were generated from the standard deviation of twenty measurements of droplet absorbance under each concentration. Calibrations are performed both before and after each lab-based test and field deployment to quantify the dialysate or pore nitrate and ensure that there was no significant signal drifting. By altering the sample-to-reagent ratio and dilution factor to match the expected nitrate levels in the soil, the upper linear range of the device was increased to 50 mM, as was done in lab-based nitrate monitoring tests. We used this calibration method to quantify all nitrate sampled in droplets, which were then converted into soil-related nitrate.

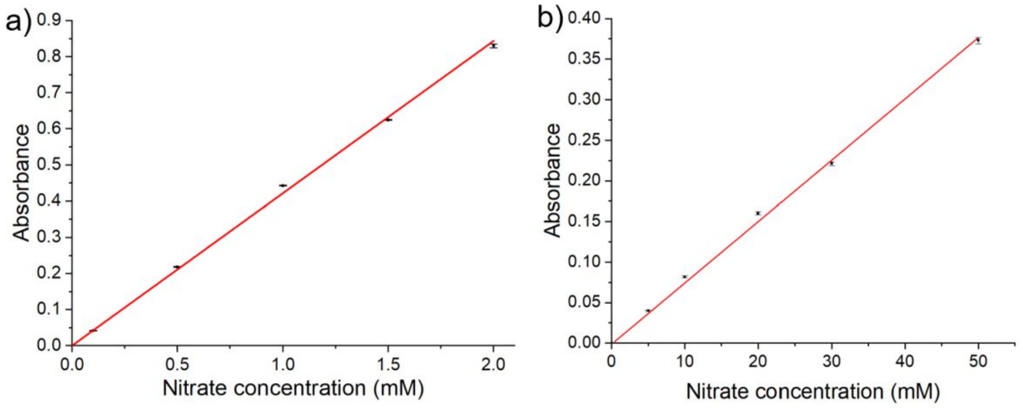

Figure S1: a) Calibration on nitrate standard solutions in the droplet microfluidic unit without the use of sampling probe under 1:1 sample to reagent ratio. b) Calibration on nitrate standard solutions in droplet microfluidic unit without the use of sampling probe under 1:2:2 sample to reagent to dilution water ratio.

Text S8: Full droplet absorbance response over time

Figure 2 displays the full droplet absorbance response data obtained from microdialysis and ultrafiltration systems after stop flow. Figure 2a and c demonstrate the corresponding absorbance changes when the flow contains previous droplets, residual sample in the probe outlet, and fresh sample transferred to the detection flow cells. Figure 2b and d provide schematics for each analyzed segment in the two systems, which are highlighted in green. The previous droplets exhibited a higher absorbance due to a longer reaction time. The residual sample consisted of nitrate with varying concentrations from the last test, while the fresh sample was nitrate sampled from the current lab-scaled soil column spiked with a standard nitrate solution.

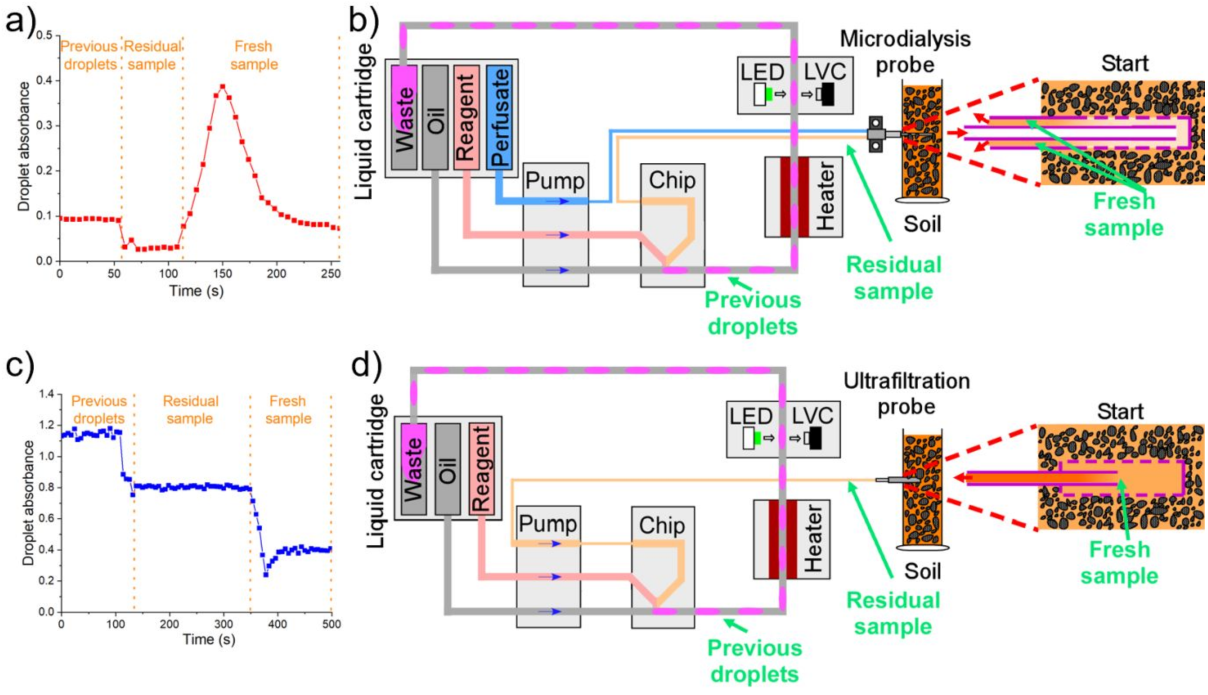

Figure S2: Raw absorbance data of each droplet measured via the detection flow cell, corresponding to previous droplet, residual sample, and fresh sample, and schematics showing where each segement flow present in the systems (a,b) from microdialysis system (c,d) ultrafiltration system.

Text S9: Soil moisture-dependent recovery under varied stop time

The soil moisture-dependent recovery of microdialysis system was found to be consistent for 6 and 12-hour stop time under different soil moisture content, with only a 7% difference in gradient (within the error of recovery). The increase of stop time from 6 to 12 hr did not show significant difference in the resulted recovery, which suggests the stop times are sufficient to build up an equilibrium between external nitrate in soil phase and the dialysate nitrate in the probe tip that diffused through the semipermeable membrane.

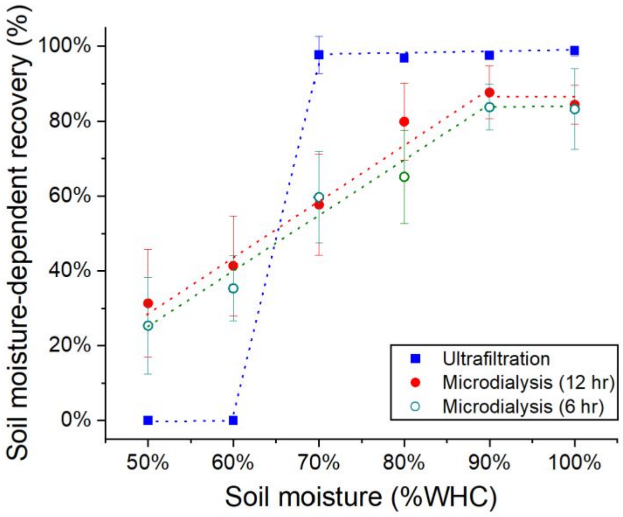

Figure S3: Change in relative recovery under different soil moisture contents from 50 to 100 %WHC, measured from either microdialysis (6 hr or 12 hr waiting time) or ultrafiltration (12 hr waiting time) coupled sensor.

Text S10: Method validation on standard soil

We measured nitrate-loaded dried soil (15.0 μg NO_3^- per g of dried soil) with different moisture contents (50-100 %WHC) to test whether our developed method is valid for estimating the absolute soil nitrate. Fig. S4a shows the measured $C_{dialysate}$ or C_{pore}. As the moisture content reduced from 100 %WHC, C_{pore} from ultrafiltration unit increased as the nitrate in solution was more concentrated when less water was added to the dried soil. On the contrary, $C_{dialysate}$ showed a gradual decrease with the decrease of moisture content, which can be explained that although the nitrate concentration in soil water increased, the connectivity of moisture within the soil decreased. As a consequence of poorer connectivity, the diffusively available nitrate to the microdialysis probe became less and hence reduced $C_{dialysate}$.

Nevertheless, Equation 1 in the main text was used to convert the measured nitrate ($C_{dialysate}$ and C_{pore}) to absolute soil nitrate (C_{soil}), as shown in Fig. S4b, using corresponding R_{soil} under soil moisture content of 50 to 100 %WHC (Fig. 3). The calculated absolute values (13.9 to 15.8 μg NO_3^- per g of dried soil) are very close to the expected standard value (15.0 μg NO_3^- per g of dried soil), with error on the calculated soil nitrate around 2-3 % for the ultrafiltration unit and 2-10 % for the microdialysis unit. Therefore, the experiment has validified the method of calculating the absolute soil nitrate. The larger error from microdialysis test is consistent with previous observations (Fig. 3) but is acceptable when compared to the errors normally seen during standard soil nitrate measurement *via* KCl extraction (typically in the range of 1-7 %).

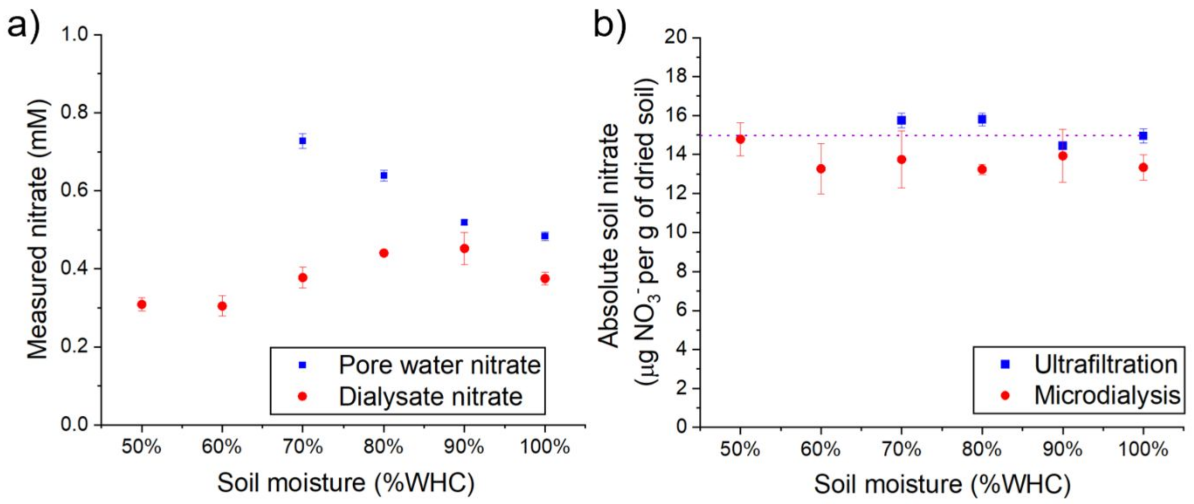

Figure S1: (a) Dialysate nitrate and pore water nitrate concentration measured from standard soil with constant absolute soil nitrate (15.0 μg NO_3^- per g of dried soil) under varied moisture from 50 to 100 %WHC. Microdialysis is in red circle and ultrafiltration data is blue square. (b) Calculated absolute soil nitrate from the measured nitrate and corresponding soil moisture-dependent recovery. The error bars correspond to the standard deviation of calculated soil nitrate from three replicates measured from three soil columns. The error bars for soil moisture for soil colunm can be neglected.

Text S11: Moisture sensor calibration

The moisture sensor that integrated on the analyzer was calibrated in lab-scaled soil columns. The soil columns were prepared in nitrate-free soil spiked with water from 50 to 100 %WHC. The normalized moisture reading was plotted againt soil moisture content in %WHC. The linear relationship was used to determine soil moisture content in Writtle forest at the sampling time. It needs to be noticed here that the maximum normalised moisture reading (at 1.0) corresponds to 120 %WHC, representing the upper detection limit of moisture sensor. When soil moisture was above the threshold, 120 %WHC was used for the calculation of absolute soil nitrate, giving the closest estimation.

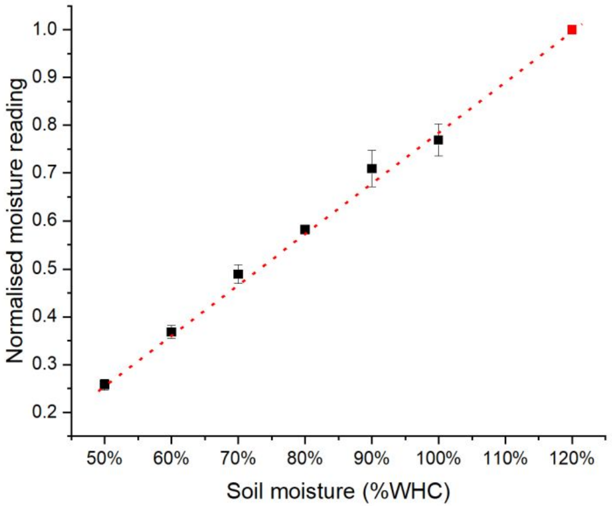

Figure S5: Moisture sensor calibration in lab-scaled soil column with varied moisture content. The error bars correspond to the standard deviation of normalised moisture reading from three replicates measured from three soil columns.

Text S12: Forecast temperature during Writtle forest deployment

Highest and lowest forecast temperature is recorded from Chelmsford weather station and plotted in the Figure 4 below over 62 days of experimental time. The lowest and highest forecast temperature fluctuated and gradually dropped down from 14th September,2022 (day 0) to 15th November, 2022 (day 62), during the seasonal transition from Summer to Autumn.

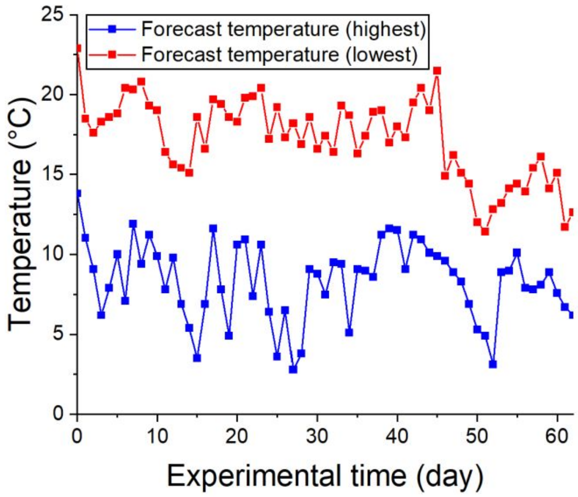

Figure S6: Forecast highest (red square) and lowest temperature (blue square) from Chelmsford weather station during the deployment in Writtle forest.
